# Received Signal Strength-Based Indoor Localization Using Hierarchical Classification

**DOI:** 10.3390/s20041067

**Published:** 2020-02-15

**Authors:** Chenbin Zhang, Ningning Qin, Yanbo Xue, Le Yang

**Affiliations:** 1 Key Laboratory of Advanced Process Control for Light Industry of Ministry of Education, Jiangnan University, Wuxi 214122, China; chenbin.zhang@foxmail.com; 2 Career Science Lab, BOSS Zhipin, Beijing 10028, China; xueyanbo@kanzhun.com; 3 Department of Electrical and Computer Engineering, University of Canterbury, Christchurch 8041, New Zealand; le.yang.le@gmail.com

**Keywords:** indoor localization, fingerprint positioning, received signal strength, hierarchical classification

## Abstract

Commercial interests in indoor localization have been increasing in the past decade. The success of many applications relies at least partially on indoor localization that is expected to provide reliable indoor position information. Wi-Fi received signal strength (RSS)-based indoor localization techniques have attracted extensive attentions because Wi-Fi access points (APs) are widely deployed and we can obtain the Wi-Fi RSS measurements without extra hardware cost. In this paper, we propose a hierarchical classification-based method as a new solution to the indoor localization problem. Within the developed approach, we first adopt an improved K-Means clustering algorithm to divide the area of interest into several zones and they are allowed to overlap with one another to improve the generalization capability of the following indoor positioning process. To find the localization result, the K-Nearest Neighbor (KNN) algorithm and support vector machine (SVM) with the one-versus-one strategy are employed. The proposed method is implemented on a tablet, and its performance is evaluated in real-world environments. Experiment results reveal that the proposed method offers an improvement of 1.4% to 3.2% in terms of position classification accuracy and a reduction of 10% to 22% in terms of average positioning error compared with several benchmark methods.

## 1. Introduction

Indoor localization refers to determining the position of an object in an indoor environment. It is an essential problem in many applications including search, rescue, and navigation in the indoor environments, monitoring and surveillance for security and defense purposes and Internet of Things (IoT) [1]. Unlike outdoor positioning, indoor positioning itself is challenging because satellite signals cannot be received reliably in indoor environments. Several localization methods have been developed [2,3,4,5,6,7,8] and they mainly involve exploiting the time difference of arrival (TDOA) [2,3], direction of arrival (DOA) [4,5] and time of arrival (TOA) [6] measurements, as well as ultra-wideband (UWB) technique [7,8]. However, these schemes require extra hardware not currently available within common mobile devices such as smartphones and tablets. Moreover, TDOA and TOA-based methods need precise synchronization among sensors or between the object to be localized and sensors, which could be practically difficult to achieve. Hence, under the constraints of low-cost deployment, we need to find different methods to solve the indoor localization problem.

Received signal strength (RSS)-based methods are common alternatives for indoor localization without requiring additional sensor modules. There have been a variety of techniques proposed for indoor positioning using RSS measurements from Wi-Fi [9], RFID [10], BLE [11], and magnetic [12] devices. Among these methods, Wi-Fi RSS-based indoor localization is of particular interests due to the following two advantages. Firstly, Wi-Fi access points (APs) have been extensively deployed in indoor environments; secondly, measuring Wi-Fi RSS is readily available in the current Wi-Fi terminals. Many regression techniques have become available for Wi-Fi RSS indoor positioning and they include the distance-based [13,14] and Gaussian Processes (GP)-based techniques [15,16]. In fact, the use of the standard log-normal model for indoor positioning (see, e.g., [17]) can be considered as a regression technique as well.

In this work, we shall take a different approach and consider the fingerprint-based indoor localization method. This is because for many practical applications, it would be sufficient if we can determine which area the object belongs to. Furthermore, the fingerprint-based approach does not require the simultaneous detection of at least three APs or knowing the precise locations of APs. Generally speaking, the deployment of a RSS fingerprinting localization system has two phases, namely the offline phase and online phase. During the offline phase, Wi-Fi RSS data at various object positions, also referred to as the reference points (RPs), are collected to build a database. Using RSS measurements as fingerprints, the object location can be estimated during the online phase. The fingerprint-based localization is essentially a pattern classification problem. Specifically, the RSS database regards the RSS measurements of the RPs as their patterns. The newly received RSS measurements are matched against these patterns to find the position estimates.

In this paper, we focus on indoor localization of an object using sparsely deployed Wi-Fi APs. This work presents a novel framework that first uses an improved K-Means clustering algorithm to partition the environment into possibly partially overlapping zones. This greatly simplifies the (offline) training and (online) localization phases at the cost of increasing the number of required classifiers. When performing the object position estimation, we use the 1NN algorithm [18] to determine which zone the object lies in and apply the support vector machine (SVM) [19] to find the exact object location estimate.

The proposed localization technique is different from existing hierarchical classification-based approaches mainly in the sense that the indoor environment of interest is automatically partitioned into possibly overlapping zones (see Section 4). This leads to reduced zone classification errors that could be costly especially for the conventional methods, because in this case, the following RP classifier would definitely produce erroneous localization output. As a result, the indoor positioning accuracy is also enhanced. The effectiveness of the new hierarchical classification-based approach was validated in a real-world hospital. Improvement of 1.7% to 3.2% in classification accuracy over direct applications of flat classification and improvement of 1.4% to 2.1% in classification accuracy against other hierarchical localization approaches were observed.

The rest of the paper is organized as follows. Section 2 introduces two types of classification frameworks and reviews some existing hierarchical localization approaches. A new hierarchical classification-based indoor localization method, including the improved K-Means clustering algorithm, SVM and one-versus-one strategy, are presented in Section 3. In Section 4, the experimental results are given. Section 5 concludes this paper.

## 2. Related Works

The fundamental assumption underlying the RSS-based indoor localization is that the RSSs at different RPs exhibit distinguishable patterns, so the fingerprint-based position determination can be achieved via classification. In this section, two main types of classification frameworks are discussed and then, we survey existing hierarchical localization approaches.

### 2.1. Flat Classification and Hierarchical Classification

**Figure 1:**
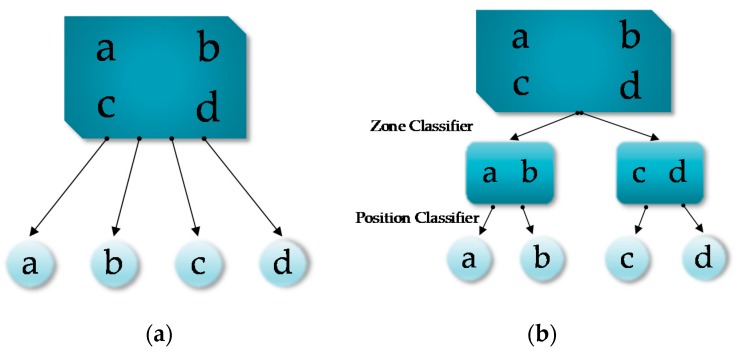
Two types of classification: (**a**) flat classification; (**b**) hierarchical classification.

The flat classification here refers to the standard binary or multi-class classification [20]. This approach directly assigns the newly obtained RSS measurements to a classification label, usually a known RP location. Figure 1a gives an illustration of such classification, where a,b,c and d represent four RPs. It can be seen that the flat classification is a one-level classification system. A flat classifier divides the input space into segments, each of which corresponds to a category (a RP in the indoor localization scenario). The KNN algorithm is a typical example of the flat classification methods and its variants have been used extensively in previous works on RSS-based location estimation. Microsoft’s RADAR [21] and Pehlavan [22] both used KNN and achieved distance errors of 2.65 m and 2.8 m respectively. In addition, SVM [23] and neural networks [24] were also used to realize the flat classification for indoor positioning. However, Sarkar [25] observed that in complex pattern classification tasks where the number of classes is large and the input space is noisy, the flat classifier may either be unable to learn correctly the pattern to class mapping or take long time to train. In other words, the flat classification may not be suitable for fingerprint-based positioning in complex indoor environments with many RPs.

Hierarchical classification refers to that the RPs are successively classified at various levels of a defined dendrogram [26]. Figure 1b gives an example, where again a, b, c and d are RPs. As we can see, the input consisting of RSS measurements from four RPs is first assigned into one of two zones, namely {a, b} and {c, d}, at the first level, and then within that zone, a RP location is output as the final localization result. In hierarchical classification, two different kinds of classifiers need to trained. Firstly, the “zone classifiers” are in charge of deciding the most likely zone that the object belongs to. Secondly, the “position classifiers” are in charge of determining the specific RP where the object is located at. The hierarchical classification follows the well-known divide-and-conquer (DAC) principle, which aims at decomposing the input space into several subspaces to enhance the learning and generalization capability. In the indoor localization problem, the hierarchy concept can be instantiated by dividing the indoor environment into several zones. Further division within zones can be applied if applicable. The hierarchical classification reduces the number of considered RPs when classifying a new measurement, thus reducing the running time as well. Therefore, for large environments or scenarios with a large number of RPs, hierarchical classification is preferred. In the next section, we shall review some previous studies on hierarchical indoor localization.

### 2.2. Existing Hierarchical Localization Techniques

The central problem of the hierarchical classification for indoor localization is how to form zones. The most common approach is to cluster the RPs. Bai [27] and Altintas [28] used the K-Means algorithm to cluster RPs, which automatically divides the entire area of interest into zones. They used the mean values of the RSS measurements to characterize each RP and perform area partition. In [27], it was indicated that using K-Means clustering could reduce the computation load and [28] showed that the clustered KNN algorithm outperforms the standard KNN method when the number of the nearest neighbors to be clustered is selected properly. Zhou [29] applied the Fuzzy C-Means (FCM) algorithm [30] to the mean values of the RSSs in order to cluster RPs into zones. In [31] and [32], Noelia and Ahmad proposed to use the AP visibility to cluster RPs when the number of RPs is large. The visibility of an AP at a RP is defined as the ratio of samples actually measured to the total number of samples intended to be collected.

**Figure 2:**
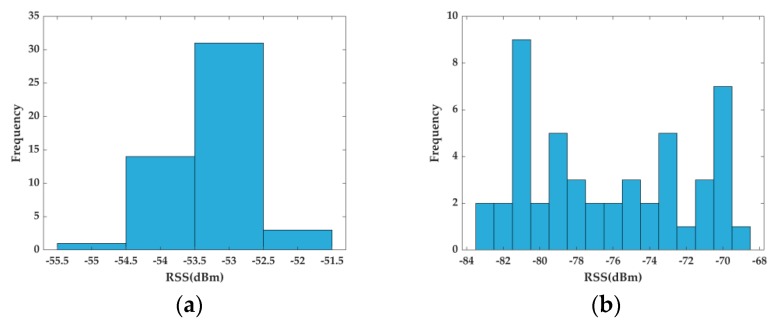
Histograms of 50 RSS samples collected at two different RPs: (**a**) RSS measurements are clustered and span a small range and it is reasonable to characterize this RP by RSS mean value; (**b**) RSS measurements spread a wider range and appear to have multiple model distribution and it is not reliable to characterize this RP by RSS mean value.

In the aforementioned studies, only a single attribute was employed to characterize a RP (either RSS mean value or AP visibility). During the establishment of the fingerprint database, usually multiple RSSs are collected at a RP. Due to the co-channel interference, multi-path effect and the absorption of wireless signals due to, e.g., walls and pedestrians, the RSS samples at a RP can fluctuate greatly and may not follow a Gaussian distribution. Therefore, exploring only the RSS mean value to characterize each RP will lose high-order statistical information in the raw data, and thus the results obtained may not be reliable. Figure 2 shows a histogram of the RSS measurements from two RPs that were collected in our experiment. From Figure 2a, we can see that for this particular RP, the RSS measurements are clustered and span a small range. Thus, it could be reasonable to use the RSS mean value to characterize this RP. However, the RSS measurements obtained at a different RP, as shown in Figure 2b, spread a wider range and appear to have a multi-mode distribution. As a result, using only the RSS mean value to represent this RP would lose much of the available statistical information. In [31] and [32], the AP visibility was employed as the attribute of a RP for clustering. But this leads to increased classification difficulty when classifying the measurements into zones, which reduces the accuracy of positioning. We shall elaborate this point more in Section 4.

## 3. System Model

**Figure 3:**
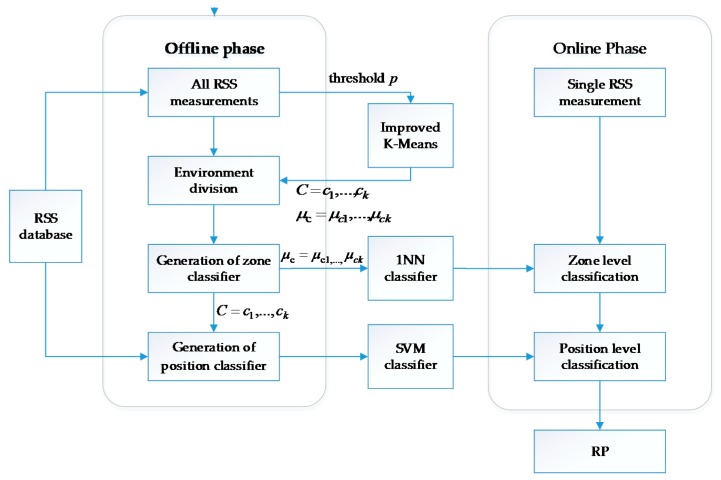
Proposed hierarchical indoor localization system.

In this section, we present a new hierarchical classification framework for Wi-Fi RSS-based indoor positioning, which again contains the offline training phase and online localization phase. The block diagram of the entire system is shown in Figure 3. For the offline phase, we propose an improved K-Means algorithm to cluster RPs. The RPs are allowed to be included in more than one clusters, i.e., the obtained clusters can overlap with one another. We then train the 1NN method as the zone classifier and SVMs as the RP position classifier. On the localization stage, newly received RSS measurements are input into the hierarchical localization system to generate the online positioning result.

### 3.1. Hierarchical Classification with Cluster Overlap

Within the conventional hierarchical classification structure, as shown in Figure 1b, zones are non-overlapping, which means that a RP can only belong to a single zone. But the RSS samples at a RP may be clustered into more than one zones. As a result, exiting hierarchical classification techniques use one attribute only to represent one RP to address this problem. However, in this case, a classification error in the zone level could be quite costly, as this means the result produced by the position classifier would be erroneous as well.

**Figure 4:**
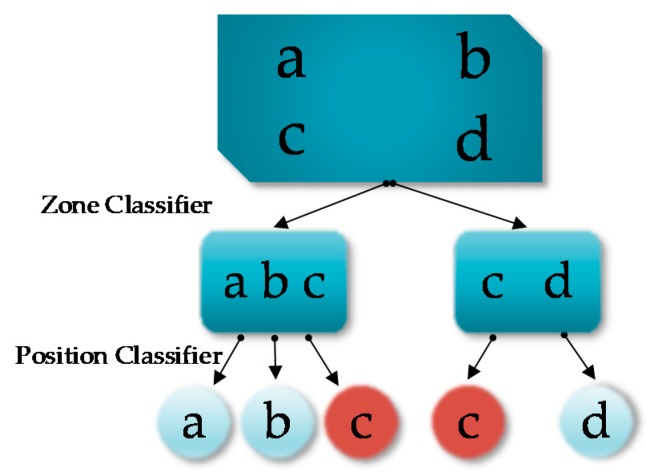
Hierarchical classification-based indoor positioning with zone overlap.

In the proposed hierarchical classification structure, zones are allowed to overlap. For illustration purpose, an example is shown in Figure 4. It can be seen that RP c belongs to two zones {a, b, c} and {c, d}. In other words, the two zones are overlapped in the position space. Since the zones could be overlapped, we refer to this classification framework the hierarchical classification with cluster overlap. This scheme can reduce zone-level classification errors, which can be better understood by noting that in Figure 4, if RP c is the true localization output, no matter whether the zone classifier outputs {a, b, c} or {c, d}, this intermediate decision is always correct. With this observation in mind, we can use a simple zone classifier while adopting more complex classification techniques in the remaining levels. On the other hand, because the zones can be overlapped, we can use all RSS samples for RP clustering, so that more statistical information in the RSS raw data can be utilized.

### 3.2. Offline Training Phase

The offline phase accomplishes three tasks: clustering RPs, training the zone classifiers and training the position classifiers. In the developed system, we use the improved K-Means algorithm to cluster RPs, 1NN algorithm as the zone classifier, and SVM as the position classifier.

#### 3.2.1. Area Partition

The area of interest is automatically divided into zones using an improved K-Means clustering algorithm. Suppose there are n RPs in the indoor environment. Let $m_{i},i=1,\ldots ,n$, denote the number of RSS measurements collected at the i-th RP. Furthermore, suppose there are N APs installed. Thus, we can express the RSS measurements obtained at the i-th RP as

(1) $$RSS_{i,j}=\left[rss_{i,j}^{1},\ldots ,rss_{i,j}^{N}\right],\;\;\;i=1,\ldots ,n;\;\;j=1,\ldots ,m_{i}.$$

Clearly, $RSS_{i,j}$ is a 1×N row vector. The entire RSS database can be represented as

(2) $$\mathrm{RSS}\;\mathrm{database}=\left\{\left\{RSS_{1,1},\ldots ,RSS_{1,m_{1}}\right\},\ldots ,\left\{RSS_{n,1},\ldots ,RSS_{n,m_{n}}\right\}\right\}.$$

The improved K-Means clustering algorithm achieves the purpose of clustering RPs by exploiting the available RSS measurements from all RPs. Specifically, this method first uses the standard K-Means algorithm to assign the RSS measurements to K clusters $C=\;c_{1},\ldots ,c_{K}$ and at the same time, find their centroids $\mu_{C}=\mu_{c_{1}},\ldots ,\mu_{c_{K}}$. Here, $\mu_{c_{k}}$ is also a 1×N vector and each entry is the mean value of a particular AP’s RSS measurements in cluster $c_{k}$. That is, it can be mathematically expressed as

(3) $$\mu_{c_{k}}=\left[rss_{\mu_{c_{k}}}^{1},\ldots ,rss_{\mu_{c_{k}}}^{N}\right].$$

Next, we determine which clusters a RP belongs to by calculating the ratio of the number of RSSs from this RP in each cluster to the total number of RSSs collected at this RP. Suppose that in cluster $c_{k}$, there are $num_{k}^{i}$ RSS measurements from the i-th RP. We then calculate the ratio $num_{k}^{i}/m_{i}$. If it is greater than a threshold p, we have that cluster $c_{k}$ contains significant amount of RSSs from the i-th RP, so the i-th RP is assigned to cluster $c_{k}$. This process is repeated for each RP. The value of p is user-defined and it controls the size of the overlapping area. The smaller the value of p is, the more zones will overlap. The improved K-Means algorithm is summarized in Algorithm 1.
**Algorithm 1:** Improved K-Means clustering algorithmInput: RSS database in (2), threshold p Output: RP’s cluster labels $C=\;c_{1},\ldots ,c_{K}$, K cluster centers $\mu_{C}=\mu_{c_{1}},\ldots ,\mu_{c_{K}}$Initialize K centroid points.Calculate the Euclidean distance between the RSS measurements and cluster centroids using $$d_{i,j,k}={\left|\left|RSS_{i,j}-\mu_{c_{k}}\right|\right|}_{2}$$Assign the RSS measurements to the cluster whose centroid is the closestWhen all RSS measurements are assigned, re-calculate the cluster centroidsIterate step 2, 3 and 4 until convergenceOutput the cluster centers $\mu_{C}=\mu_{c_{1}},\ldots ,\mu_{c_{K}}$Compute $num_{k}^{i},i=1,\ldots ,n,\;k=1,\ldots ,K$If $\frac{num_{k}^{i}}{m_{i}}>p$, assign the i-th RP to cluster $c_{k}$When all RPs are assigned, terminate.

This clustering method uses all the RSS measurements for clustering and the final clustering results are expressed in terms of each RP belonging to at least one clusters, which accomplishes the area partition. It is worth mentioning that the value of p should satisfy

(4) $$p\in (0,\frac{1}{K}].$$

The justification for the lower limit of p is straightforward. In words, p>0 means when the number of RSS measurements from $RP_{i}$ in a cluster $c_{k}$ is zero, the $RP_{i}$ should not be included in zone $c_{k}$. The justification for the upper limit of p in (4) is as follows. It can be shown that

(5) $$\{\begin{array}{c} \overset{K}{\underset{k=1}{\sum }}\frac{num_{k}^{i}}{m_{i}}=1\\ num_{k}^{i}\geq 0\\ num_{k}^{i}\leq m_{i} \end{array}.$$

In order to ensure that the RP would be clustered into at least one zone, it is required that

(6) $$\underset{k}{\max}\frac{num_{k}^{i}}{m_{i}}>p,k=1,\ldots ,K.$$

Therefore, for all n RPs, to guarantee that each RP is clustered into at least one zone, the value of p should also fulfill

(7) $$\underset{i}{\min}\;\underset{k}{\max}\frac{num_{k}^{i}}{m_{i}}>p,i=1,\ldots ,n,k=1,\ldots ,K.$$

Because $num_{k}^{i}$ cannot be obtained before the clustering algorithm is terminated, p should be set to a value such that (7) is satisfied even in the worst case. This value can be found via solving the following minmax optimization problem

(8) $$\min\underset{k}{\max}\frac{x_{k}}{m_{i}},k=1,\ldots ,K\;\;\mathrm{subject}\;\mathrm{to}\;\;x_{k}\geq 0\;x_{k}\leq m_{i}\;\overset{K}{\underset{k=1}{\sum }}\frac{x_{k}}{m_{i}}=1$$

The solution can be found to be

(9) $$\frac{x_{1}}{m_{i}}=\frac{x_{2}}{m_{i}}=,\ldots ,\frac{x_{K}}{m_{i}}=\frac{1}{K}.$$

The upper limit for p is now verified.

#### 3.2.2. Zone Classifier

In the developed indoor localization system, we use the 1NN algorithm, a special case of the KNN method with K=1, as the zone classifier. KNN is one of the simplest machine learning algorithms. It classifies an object using a voting mechanism which selects the most voted class among the k neighbors closest to the object. The KNN was used in RADAR which is widely recognized as one of the pioneering works in the field of Wi-Fi indoor localization and usually considered as a baseline to evaluate new indoor Wi-Fi localization systems [31]. We use 1NN as the zone classifier for the following reasons: (1) The presence of zone overlap means that RSS data in different zones could the same, which is not appropriate for the classification models that require pre-training. (2) The classification difficulty of the first level is relatively low, especially under the overlapped area partition. Therefore, 1NN would be sufficient for the first-level zone classification.

We use the RSS measurement cluster centers as the first level database and use the 1NN to classify the zones. When a new set of RSS measurements are obtained, we calculate the Euclidean distance between these measurements and each cluster center $\mu_{c_{1}},\ldots ,\mu_{c_{K}}$. The cluster $c_{k}$ with the closest center $\mu_{c_{k}}$ is selected as the zone for the newly collected RSS measurements.

#### 3.2.3. Position Classifier

**Figure 5:**
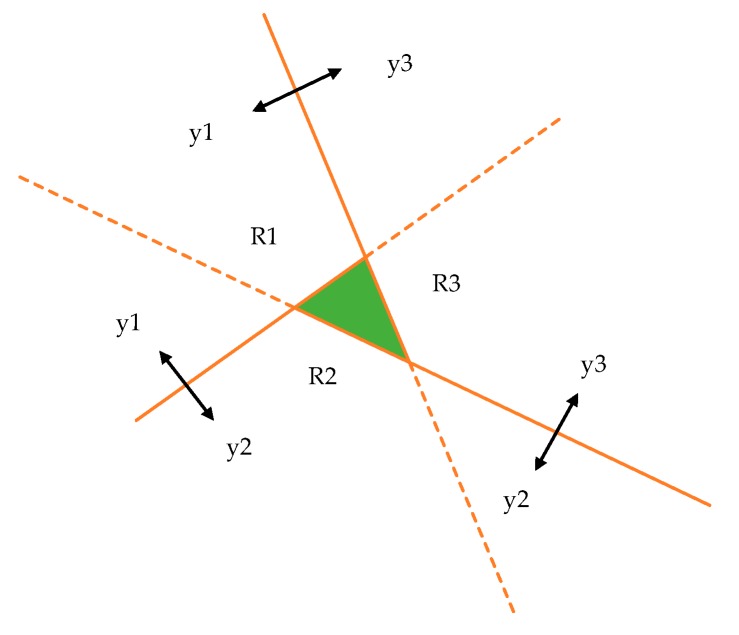
Ambiguities in the one-versus-one approach. The green area represents the ambiguous part.

We proceed to train position classifiers for each zone for position determination. The SVM is used as the position classifier to distinguish RPs in a zone through exploiting all the RSS measurements. The basic form of SVM is implemented for binary classification problem for linearly separable datasets. For the nonlinear case, the kernel trick maps the feature space to make the data linearly separable in the high-dimensional space, enabling the SVM to handle nonlinear classification problems. For multi-class classification, a number of SVM-based binary classifiers are required to distinguish multiple classes. The one-versus-all [33] method and the one-versus-one method [34] are two commonly adopted techniques to solve multi-class classification problems using binary classifiers. In [23], the authors proved that the one-versus-one method performs better in indoor localization. So, in our system, we use the one-versus-one SVM to solve the position classification problem. In a N-class problem, the use of the one-versus-one strategy requires training N(N−1)/2 binary SVM classifiers to discriminate all RP pairs. The final decision is selected to be the RP with the highest number of votes. However, the one-versus-one approach may result in regions of the position space which are ambiguously labeled as shown in Figure 5. Among the N(N−1)/2 votes, if two or more labels receive the largest votes at the same time, there will be localization ambiguity. In our system, if the localization ambiguity occurs, the selected zone would be taken as the positioning result. We call this operation “back to zone”. In other words, in this case, we choose to enlarge the positioning area to obtain a more reliable localization result. This method is a compromise between the positioning accuracy and reliability.

### 3.3. Online Phase

In the online localization phase, we cascade the trained zone and position classifiers, forming a complete hierarchical localization system. When new RSS measurements are input into the system for position determination, the system first uses the 1NN zone classifier to determine the zone where the target is located. After determining the zone, the system inputs the RSS measurements into the SVM position classifier corresponding to the particular zone to obtain the estimated position of the target.

## 4. Experiments and Results

**Figure 6:**
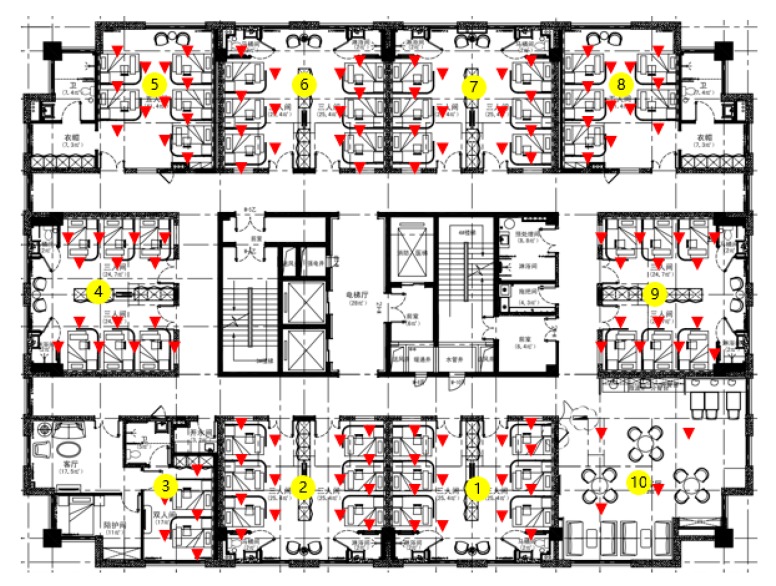
The schematic diagram of the test environment, where the rooms and RPs are labeled with yellow circles and red triangles, respectively.

In order to evaluate the proposed hierarchical classification scheme in a real-world environment, we conducted a measurement campaign in a hospital in Wenling, Zhejiang, China. Since the patients in this hospital are most elderly, the accuracy in both zone classification and position determination plays a vital role in emergency handling. As shown in Figure 6, the test environment is a 36 m × 26.8 m rectangular floor where 4 Wi-Fi APs were deployed. There are ten rooms in this floor, and they are numbered from 1 to 10. When collecting the Wi-Fi RSS measurements, we selected 108 RPs and used a tablet to collect 50 RSSs for each RP. The Wi-Fi RSS detection range of our tablet is from −5 dBm to −95 dBm. For some RPs, less than 4 RSS measurements were obtained. To address this problem, we set the RSSs of the undetected APs to be −100 dBm.

In our offline training phase, the RSS database was divided into two parts, with 50 percent of the measurements put into the training dataset and the remaining data being in the test dataset. The performance of our method is compared with several baseline algorithms, including SVM, KNN, multilayer neural network (NN), K-Means [27], Visibility [31], and FCM [29]. As mentioned in Section 2, FCM evaluates the probability of a measurement belonging to a certain zone using a fuzzy algorithm, which could be considered as a hierarchical classification method. We improved the original FCM technique by adjusting its membership degree threshold to allow one RP to be clustered into multiple clustering categories (i.e., zone overlap is allowed).

For the benchmark methods and the proposed algorithm, four performance metrics are considered for comparison. They include the zone classification error rate, position classification accuracy, average positioning error and running time. The zone classification error rate is the percentage of the number of test RSS measurements assigned to zones that do not contain their true RPs. The position classification accuracy gives the percentage of test RSS measurements assigned to the correct RPs. The average positioning error is the average distance between the selected RPs for the test RSS measurements and their true RPs.

### 4.1. Area Division Result

**Figure 7:**
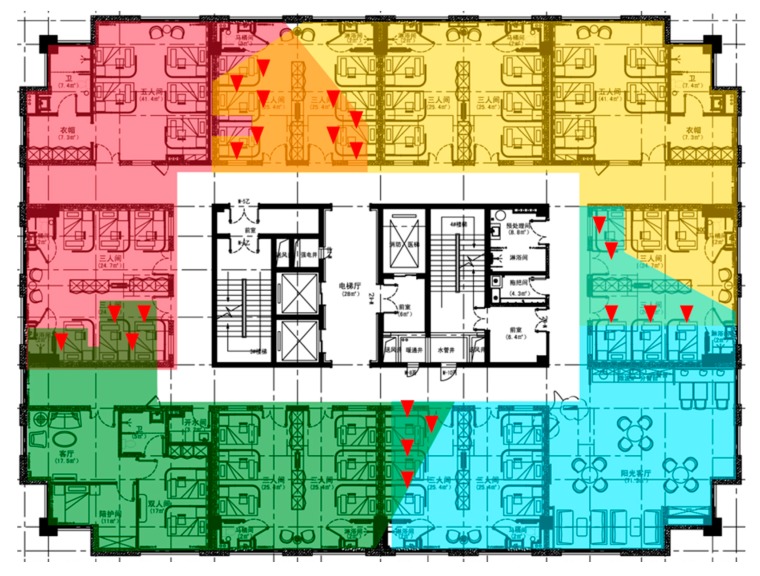
Area partition result. In total, 4 zones are formed after clustering, which are depicted in red, yellow, green and blue. In total, 22 RPs denoted by red triangles are located in the overlap regions.

Since the experimental environment is a rectangle and 4 Wi-Fi APs have been installed priorly, it can be naturally divided into 4 parts, which makes our choice of K in Algorithm 1 to be 4. Results of the improved K-Means clustering method are depicted in Figure 7, where four zones are depicted in red, yellow, green and blue. The soft labelling nature of our algorithm is reflected by the existence of 4 overlapped areas with 22 RPs, which reduces the difficulty of zone classification and propagates more information rather than errors to the position classification level.

The step that evaluates $num_{k}^{i}/m_{i}>p$ in Algorithm 1 is a hard-thresholding process and the value of p determines the size of the overlapped areas. When the numbers of RSS measurements from a RP in all clustering categories are less than p, the RP would be left out. Therefore, in order to ensure that all the RPs are assigned into at least one zone, the value of p must be in the suggested range given in Equation (4).

**Figure 8:**
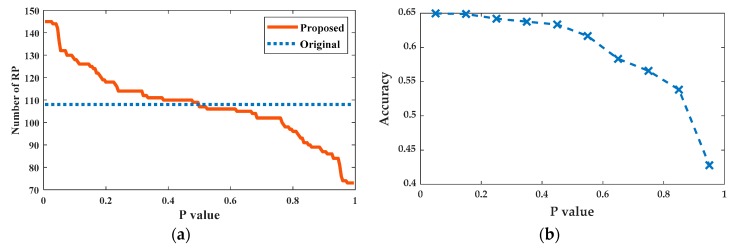
(**a**) Total number of RPs counted in the 4 zones generated using the proposed improved K-Means clustering algorithm. The dotted blue line shows the true RP number for comparison. (**b**) Position classification accuracy as a function of p.

Figure 8a plots the total number of RPs in the four formed zones as a function of p. When p is small, the total number of RPs in four generated zones would be larger than the true RP number because the RPs in the overlap regions are counted more than once. With the increase of p, more RPs become left out, and the total number of RPs in the four zones decreases accordingly. When p is greater than 0.5, the total number of RPs in the four zones is less than the number of original RPs, indicating that there must be some RPs not assigned to any zones.

In Figure 8b, we plot the position classification accuracy of the proposed localization method as a function of p. It can be seen that the position classification accuracy deteriorates marginally when p is less than 0.2 but significant performance degradation occurs after p increases over 0.5. The underlying reason is that when p is small, the increase in its value reduces slightly the size of the overlapped areas only, which would not greatly enlarge the zone classification error rate and as a result, the position classification accuracy does not decrease evidently. On the other hand, when p is sufficiently large, a few RPs would start to be left out. This increases the zone classification error rate and leads to degraded position classification accuracy.

### 4.2. Zone Classification Accuracy

An error in the zone classification stage will be propagated to and even amplified at a later stage under the hierarchical classification structure, which makes the accuracy of zone classification a very important performance indicator. We use 1NN as the zone classifier to compare the zone classification error rates of four hierarchical classification methods in consideration and the results are shown in Table 1. As we can see, the FCM has the best performance with a zone classification error rate of 1%, followed by our proposed method with an error rate of 2.25%. FCM and the proposed method are both hierarchical classification approaches with zone overlap. This implies that allowing zone overlap when forming clusters using RSS measurements can effectively reduce the difficulty of the zone classification.

With the hierarchical classification with overlap, the accuracy of zone classification improves with the increase in the overlap regions. When the overlap region is large as the test environment, the number of zones reduces to one and the zone classification error rate will become zero, which renders the hierarchical classification meaningless. Actually, when the overlap regions are too large, the efficiency of the hierarchical classification will dramatically decrease as well (see Section 4.3). In FCM, there are 70 RPs in the overlap regions, compared to 22 RPs in our method. This manifests as a higher zone classification accuracy of FCM. In the subsequent experiments, we shall further investigate the effect of overlap area size on the position classification accuracy and average positioning error.

### 4.3. Positioning Accuracy

In our experiment, the direct use of KNN, multilayer NN and SVM for flat classification-based object position estimation are selected as the benchmarks for performance evaluation. Specifically, the multilayer NN has 8 fully-connected hidden layers with 10, 18, 27, 35, 22, 16, 15 and 22 neurons, respectively, and they adopt the tanh activation function. The cross-entropy loss is employed in the training process, where 85% of the training data is used for optimizing the connection weights while the remaining 15% is applied for cross validation to avoid overfitting. In addition, we simulate three hierarchical localization methods, namely K-Means [27], Visibility [31] and FCM [29]. Similar to the proposed technique, they adopt 1NN as the zone classifier and SVM as the position classifier. But they have different zone classification schemes (see Section 2 for a survey). We conduct 5 randomized experiments, each with different RSS raw data selected to generate the training set and test set.

The averaged positioning performance results of the methods in consideration are summarized in Table 2. The results reveal that the positioning performance of our method is better compared with other methods, reaching the highest position classification accuracy of 64.97% and the lowest average positioning error of around 1.29 m. Even though the improvement in terms of the position classification accuracy appears to be small (only 1.4%–3.2%), the proposed technique leads to at least 10% decrease in the average positioning error over other hierarchical classification-based methods while it offers at least 15% reduction in the average positioning error compared with flat-classification algorithms. The reason behind is that with improvement in the position classification accuracy, more RSS measurements would be assigned to their true RPs, which would greatly decrease the average positioning error as in this case, we have more cases with a positioning error of 0 m.

Another observation is that the performance of the multiplayer NN is inferior to those of the proposed method and even SVM. The underlying reasons could be as follows. (1) The amount of RSS measurements collected for the experiment may not be sufficient for the training of the multilayer NN to converge to a local optimum with good generalization capability. (2). The hierarchical nature of the indoor localization problem is not explored by the multilayer NN but it is explicitly accounted for in the development of the proposed method. Increasing the volume of the training dataset could be useful to obtain a multilayer NN with significantly enhanced performance and this would be subject to future measurement campaign and experiments.

It is worth mentioning that the zone classification error rate of the FCM-based method is the lowest among the considered techniques (see Table 1), but its positioning performance is not the best. This is because (1) it generates regions with large overlap and this improves the accuracy of zone classification, but also makes the zone classification result unable to provide sufficient information for the following position determination; and (2) FCM still uses the mean values of the RSS measurements to characterize each RP, so it fails to explore the high-order statistical information in the raw data.

**Figure 9:**
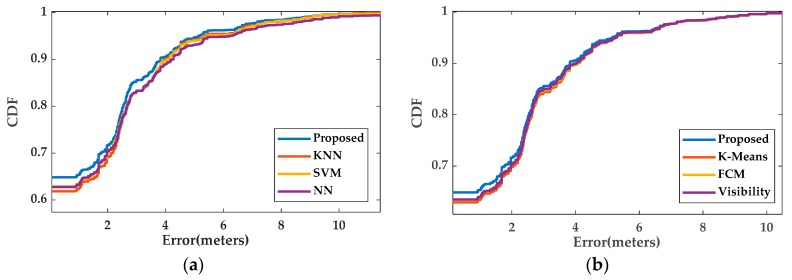
CDFs of the positioning errors from the benchmark and proposed localization techniques in consideration: (**a**) comparison with the flat classification methods; (**b**) comparison with the hierarchical classification methods.

In Figure 9, the cumulative probability distributions (CDFs) of the positioning errors from the benchmark methods and the proposed technique are depicted. Note that the values of the CDF curves at the positioning error of 0m correspond to the position classification accuracy. Again, the enhanced localization performance of the proposed algorithm over the existing techniques is evident.

### 4.4. Running Time

The running time is a key performance metric for an online system. We evaluate the running time of all the considered algorithms in the previous subsection. The obtained results are shown in Table 3. Our test environment has 108 RPs. A direct use of SVM with the one-versus-one strategy requires 108×107/2=5778 SVM binary classifiers, or equivalently a running time of 5.1 s. Compared with other methods, KNN and NN have the smallest running time, where the running time of KNN is only 0.08 s. However, due to the difficulty in finding efficient classification hyperplanes, their indoor positioning performance is inferior to the proposed technique. With the help of the hierarchical classification, the running time for one localization operation is reduced to less than 1 s, among which the K-Means requires the smallest amount. Due to the overlapped regions, the test environment is virtually expanded, and the running time is slightly longer than that of the hierarchical classification without zone overlap, as expected. However, our method still needs 0.77 s only in addition to significantly improved positioning accuracy, which achieves a better balance between the computational efficiency and performance.

## 5. Conclusions

In this paper, we presented a novel framework for the Wi-Fi RSS-based indoor localization based on hierarchical classification. Different from existing methods, we proposed to avoid disjoint partitioning and split the indoor environment into zones with possible overlap. In order to construct the category hierarchy, we proposed an improved algorithm based on the K-Means algorithm, which automatically divided the indoor region into overlapped zones according to the clustered RSS measurements. The 1NN and SVM were adopted to realize the zone and position classifications to establish the whole indoor localization procedure. The proposed method has been implemented on a tablet and evaluated in a real-world hospital under practical conditions with people moving around and simultaneous operations of electrical devices. The experimental results reveal that compared with the existing flat classification methods and hierarchical classification methods, our method has greatly reduced computation load and improved localization accuracy.

## Figures and Tables

**Table 1:** Zone classification error rate comparison.

Method	Proposed	FCM	Visibility	K-MEANS
**Zone Classification Error Rate**	2.25%	**1%**	9.07%	3.08%

**Table 2:** Positioning performance comparison.

Method	Position ClassificationAccuracy	Average Positioning Error	90th Percentile on the Positioning Error
KNN	61.76%	1.67 m	6.58 m
NN	63.05%	1.53 m	6.62 m
SVM	63.24%	1.52 m	6.65 m
Proposed (1NN+SVM)	**64.97%**	**1.29 m**	**6.33 m**
K-Means (1NN+SVM)	62.83%	1.54 m	6.51 m
Visibility (1NN+SVM)	63.49%	1.46 m	6.45 m
FCM (1NN+SVM)	63.45%	1.44 m	6.42 m

**Table 3:** Running time comparison.

Method	SVM	Proposed (1NN+SVM)	K-Means (1NN+SVM)	Visibility (1NN+SVM)	FCM (1NN+SVM)	KNN	NN
Running Time	5.1s	0.77s	0.72s	0.82s	0.97s	**0.08s**	0.22s
